# Trends and Causes of Neonatal Mortality in Serbia, 1997-2016

**DOI:** 10.4274/balkanmedj.galenos.2020.2019.5.145

**Published:** 2020-04-10

**Authors:** Konstansa Lazarević, Dragan Bogdanović, Ljiljana Stošić

**Affiliations:** 1 Department of Biomedical Sciences, State University of Novi Pazar, Serbia; 2 Nis University School of Medicine, Serbia

**Keywords:** Causes, neonatal mortality, Serbia

## Abstract

**Background:**

Regardless of the notable reduction in infant deaths worldwide over the last 30 years, the proportion of neonatal mortality in total child mortality is increasing.

**Aims:**

To perform a trend analysis of neonatal mortality in Serbia.

**Study Design:**

Descriptive observational study.

**Methods:**

Joinpoint regression was used to analyze neonatal mortality data for the years 1997 to 2016 that were obtained from the Statistical Office of Serbia.

**Results:**

The trend in the neonatal mortality rate decreased significantly by -5.6% (95% CI: -6.5 to -4.6) per year from 1997 to 2007, and by -2.6% (95% CI: -3.7 to -1.5) per year from 2007 to 2016. The neonatal mortality rate for certain conditions originating in the perinatal period decreased by -6.2% (95% CI: -7.5 to -4.9) per year during the years from 1997 to 2006, and by -1.9% (95% CI: -3.1 to -0.7) per year from 2006 to 2016. Among these conditions, disorders related to short gestation and low birth weight, not elsewhere classified, showed an upward trend by 8.5% (95% CI: 6.2 to 10.8) per year during the entire study period. From 1997 to 2016, a significant decrease in the neonatal mortality rate was detected in cases of congenital malformations, deformations, and chromosomal abnormalities, by -5.0% (95% CI: -6.1 to -4.0) per year. The neonatal mortality rate for cases of symptoms, signs, and abnormal clinical and laboratory findings, not elsewhere classified, decreased by -8.1% (95% CI: -11.0 to -5.2) yearly.

**Conclusion:**

The neonatal mortality rate in Serbia decreased between 1997 and 2016, excluding deaths due to short gestation and low birth weight. Therefore, prevention of short gestation and low birth weight should be the highest public priority.

From 1990 to 2015, the implementation of the Millennium Development Goals (Goal 4) had the objective of decreasing world mortality of children under the age of 5 years (93 deaths per 1000 live births) by two-thirds. At the end of these 15 years (2000–2015), the mortality rate of children under the age of 5 years was almost halved (43 deaths per 1000 live births), whereas neonatal mortality also decreased (from 33 deaths to 19 deaths per 1000 live births). However, the share of neonatal deaths in the mortality rate of children under five years old showed an upward trend, from 36% in 1990 to 44% in 2015 (1).

The reported decline in neonatal mortality is insufficient when we consider great variations in the rates and causes of neonatal mortality between regions and countries. Southern Asia and sub-Saharan Africa are world regions with the highest neonatal mortality rates (NMRs), with more than three-quarters (79%) of the world’s neonatal mortality. Among the countries with the highest NMRs are Pakistan (46 deaths per 1000 live births), while the lowest are Iceland and Japan (one death per 1000 live births) (2). In sub-Saharan Africa and South Asia, infectious diseases account for approximately one-quarter of all neonatal deaths. In contrast, in high-income countries, they account for only 7 percent of neonatal deaths (3).

The Republic of Serbia is an upper-middle-income country, located in Southeast Europe (Balkan Peninsula). According to the latest 2011 census, Serbia had a population of 7,186,862 inhabitants, of which almost 60% live in urban areas. More than 1,600,000 inhabitants, or 23% of the total population of the Republic of Serbia, live in Belgrade, the country's capital. Between the two censuses, 2002 and 2011, the population decreased by 4% (311,139 inhabitants) (4).

Serbia ranks among the European countries with the oldest population. The aging index (the number of people over the age of 60 per 100 youths under the age of 19) was 96.44 in 2000 and 139.54 in 2016. Also, the total mortality rate (per 1000 people) increased from 13.84 in 2000 to 14.29 in 2016, while the live birth rate decreased from 9.81 in 2000 to 9.17 in 2016. The number of females in the age group of 15–49 years was reduced from 24.4% in 2000 to 22% of the total population in 2016 (5,6).

As a political response to this negative demographic trend in Serbia, and to encourage women to have children, in 2002, the Law on Financial Support for Families with Children was introduced. It was amended later in 2005 and 2009. This Law was financed from the national budget and regulated child benefits, parental allowance, maternity leave payments, and leave of absence payments for the special care of children. Nevertheless, in 2016, fertility rates in Serbia were still low (1.46), and only 15.6% of live-born children were the third or higher in their birth order (5).

Beginning in December 2017, Serbia has been applying the new Law on Financial Support for Families with Children to decrease the financial burden of parenthood and reconcile the one posed by working and parenting (2017). The amendment of this Law beginning on July 1, 2018, significantly increased parental allowances, especially for the third or fourth child (7).

Since the start of the 21st century, mortality rates of children under the age of five years in Serbia decreased more than half (from 13.0 in 2000 to 6.0 per 1000 live births in 2016) (8). Although the NMR also dropped from 7.8 in 2000, to 4.0 per 1000 live births in 2016 (8), the contribution of neonatal mortality to mortality under 5 years of age increased from 60.4% (567 of 939 deaths) in 2000 to 65.2% (257 of 394 deaths) in 2016 (5,6).

The aim of this nationwide study was to analyze the mortality trends and causes of neonatal deaths in Serbia during the period from 1997 to 2016. These results may help to identify groups of newborns who require specialized healthcare and recognize risk factors for neonatal mortality.

## MATERIALS AND METHODS

This observational descriptive surveillance system evaluation study included all live-born children in Central Serbia and the Province of Vojvodina for the years 1997–2016, excluding the Province of Kosovo and Metohija (data unavailable), which declared itself independent in 2008. This study was approved by the Ethics Committee of Public Health Institute, Nis (number 07-1208). Annual data on all live births and neonatal deaths were obtained from the Statistical Office of the Republic of Serbia. In Serbia, nearly 99% of births are registered. They occur in health institutions and with medical attendance (9,10).

Neonatal death is defined as the death of a newborn occurring during the first 28 days of life (0–27 days). Early neonatal mortality occurs during the first seven days of life (0–6 days), whereas late neonatal mortality occurs after the seventh day of life but before the 28^th^ day of life (7–27 days) (11). The International Classification of Diseases, 10^th^ (ICD-10) revision was applied to classify the diagnosis of causes of neonatal deaths.

### Statistical Analysis

We used descriptive statistics (numbers, percentages, and rates) to indicate the share of causes of death according to gender in total neonatal mortality. A chi-square test was performed to compare NMRs between males and females. Data were classified as per the diagnosis of neonatal death and analyzed regarding annual NMRs per 1000 live-born children and the annual percent change (APC) in NMRs. NMRs were calculated as the number of neonatal deaths per 1000 live births (11).

The trends in NMRs were analyzed using the joinpoint regression model. This analysis fits a series of straight lines (time periods) on a log scale to the NMRs and detects the points in time (joinpoints) where significant changes in the trend occur, using NMR as the dependent variable and calendar year as the independent variable. The optimal number of joinpoints was identified using the Monte Carlo permutation method, starting with zero joinpoints and testing whether more joinpoints must be added to the model. APCs in NMRs and the corresponding 95% confidence intervals are computed for each defined period using a generalized linear model assuming a Poisson distribution. P-values <0.05 were considered significant. The average annual percentage change was also calculated for the 1997–2016 period using the average of the APCs from the joinpoint model (12). Joinpoint analyses were effectuated using the Joinpoint Regression Program version 4.2.0.2. (Statistical Methodology and Applications Branch, Surveillance Research Program, US National Cancer Institute).

## RESULTS

In the 20 years in the Republic of Serbia, there were 1,437,631 births, of which 1,430,005 were live-born children (737,854 males and 692,151 females). Of these, 8431 (0.6%) of newborns died during the neonatal period (4985 males, or 59.1% and 3446 females, or 40.9%).

**Figure 1:**
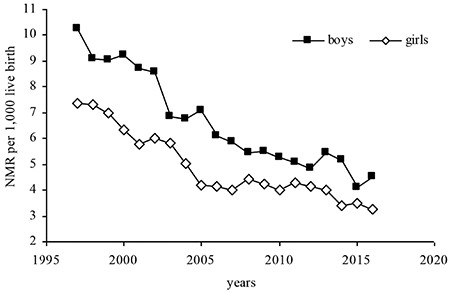
Trend in neonatal mortality rate per 1000 live births in Serbia, 1997–2016, by gender. NMR: neonatal mortality rates

During the 1997–2016 period, the NMR decreased from 10.2 per 1000 live births to 4.5 among males, and from 7.3 to 3.3 among females. Overall, males had a somewhat higher NMR when compared with females (Figure 1). Figure 1 shows the trend in the NMR per 1000 live births in Serbia, 1997–2016, by gender.

The total NMR decreased more than two-fold, from 8.8 in 1997 to 3.9 neonatal deaths per 1000 newborns in 2016. The joinpoint analysis defined two segments in the APC trend. The first period, from 1997 to 2007, showed a significant decline in the NMR, with an APC value of -5.6% (95% CI: -6.5 to -4.6%). In the second period, from 2007 to 2016, the NMR significantly decreased with an APC value of -2.6% (95% CI: -3.7 to -1.5) (Table 1).

The values of the NMR for the early neonatal period had two-segmented trends. The first period, from 1997 to 2006, showed a significant decline in the early NMR, with an APC value of -6.5% (95% CI: -8.1 to -4.8%). In the second period, from 2007 to 2016, the NMR significantly decreased with an APC value of -2.8% (95% CI: -4.2 to -1.3%). During the entire 1997–2016 period, the late NMR showed a significant decline in NMR, with an APC value of -3.2% (95% CI: -4.1 to -2.3%) (Table 1). Table 1 shows the changes in the early NMRs (0–6 days of life) and late NMRs (7–27 days of life) per 1000 live births in Serbia, 1997–2016.

Certain conditions originated in the perinatal period (77.6%); congenital malformations, deformations, and chromosomal abnormalities (15%); and symptoms, signs, and abnormal clinical and laboratory findings not elsewhere classified (5.3%) accounted for approximately 98% of neonatal deaths. The distribution of neonatal deaths by causes of death among males and females was not significantly different (Table 2). Table 2 shows the distribution of neonatal deaths by causes of death and gender.

The majority of neonatal deaths were caused by respiratory distress syndrome (P22.0; 1737 deaths or 20.6%), disorders related to short gestation and low birth weight, not elsewhere classified (P07; 1612 deaths or 19.1%) and birth asphyxia (P21; 1110 or 13.2%). Allocation of the three most frequent causes of neonatal deaths was not significantly dissimilar for both males and females (Table 3). Table 3 shows the distribution of the main causes of neonatal deaths according to gender.

In the 1997–2016 period, NMR for certain conditions originating in the perinatal period decreased with average APC of -4% (95% CI: -4.8 to -3.1) (Table 4). Joinpoint analysis defined two-segmented trends in NMR conditions originating in the perinatal period changes. During the first period (1997−2006), NMR showed a significant decline with the APC value of -6.2% (95% CI: -7.5 to -4.9). In the second period, from 2006 to 2016, NMR decreased three times, with the APC value of -1.9% (95% CI: -4.2 to -1.3%).

The NMR associated with congenital malformations, deformations and chromosomal abnormalities, and NMR associated with symptoms, signs and abnormal clinical and laboratory findings, not elsewhere classified, showed a decreasing trend throughout all of the study years (APC: -5.0; 95% CI: -6.1 to -4.0 and APC: -8.1; 95% CI: -11.0 to -5.2, respectively). NMR associated with all other causes of neonatal mortality, according to ICD-10, also showed a decreasing trend (APC: -5.1; 95% CI: -8.1 to -2.1). Table 4 shows the changes in NMR per 1000 live births according to the causes of death.

During the 1997–2016 period, NMR values decreased for respiratory distress syndrome (P22) by -11.6% (95% CI: -13.2 to -9.9), and for birth asphyxia (P21) by -6.2% (95% CI: -7.6 to -4.8). NMR values for disorders related to short gestation and low birth weight, not elsewhere classified (P07), increased from 0.6 to 1.9 deaths per 1000 live births, with an APC of 8.5% (95% CI: 6.2 to 10.8) (Table 5). Table 5 shows the changes in NMR per 1000 live births according to the three most common causes of death.

## DISCUSSION

Despite the considerable progress in reducing total neonatal mortality in Serbia in the last 20 years, the rate of neonatal mortality remains high (3.9 per 1000 newborns) when compared with other European countries (1.4 per 1000 newborns in Slovenia, 1.9 in Spain, 2.8 in Greece), including neighboring countries (Croatia, Montenegro, Hungary (3.0; 2.0; 2.5 per 1000 newborns). Similar to Serbia, NMRs in Bulgaria and Romania are 3.9 per 1000 newborns for both countries (13).

In Serbia, the total NMR was higher among males than females. However, the distribution of neonatal causes of death by gender was not significantly different, and preterm birth and congenital anomalies were among the leading causes of neonatal mortality, as noted in other studies from high-income countries (14). During our study period, the diagnosis of neonatal death in Serbia improved, and there were fewer causes of neonatal deaths related to conditions without a classifiable diagnosis (R00-R99).

Starting in 2005, the Republic Public health Institute of Serbia, with its network of 24 local Public Health Institutes, began organizing the procedure for coding the causes of death in the Republic of Serbia (15). Changes in the trend of early NMR in 2006 might be an effect of these changes in reporting deaths in Serbia, as well as changes in the trend of the total NMR in 2007.

Unfortunately, NMRs from disorders related to short gestation and low birth weight in Serbia have shown an increasing trend. Mortality linked to preterm births presents a severe public health issue and contributes 35% to global neonatal mortality (2). The worldwide, preterm birth rate was 10.6% in 2014 (16). Premature birth interrupts normal fetal lung development (17), so it is not surprising that respiratory distress syndrome (18,19) and birth asphyxia (20,21) remained important causes of mortality. In our study, one-third (33.8%) of the causes of total neonatal deaths were respiratory distress syndrome (20.6%) and birth asphyxia (13.2%). According to the WHO data, the percentage of premature births in Serbia increased from 7.23% in 2000 to 11.97% in 2014 (22). Data from a few studies conducted in Serbia indicated the existence of some of the premature birth risk factors, such as maternal age (23), smoking (24), obesity, and overweight mothers (25).

Delayed maternity is often present among women in Serbia, which increases the risk of preterm delivery. In Serbia, mothers in the age group of 35 years or older gave birth to 7.7% of all live newborns in 2000, while in 2016, this percentage surged to 17.2%. In 2000, the mean age of mothers of live-born children was 26.5, and in 2016, it increased to 29.6 years (5).

Krstev et al. (26,27) observed that smoking during pregnancy in Serbia was two- to three times higher than that in the most affluent western countries, which reduced birth weight, birth length, and head circumference of newborns, and increased the risk for low birth weight. In a study conducted in five countries of Central and Eastern Europe (Czech Republic, Hungary, Romania, Slovakia, and Ukraine), smoking, preeclampsia, hypertension, and body mass index were identified as risk factors for the occurrence of preterm births (28). Results from the study by Rudic-Grujic show that every fifth pregnant woman in this study was overweight or obese before pregnancy (29).

Recent studies worldwide showed that after the use of assisted reproductive technologies (ART), women have a significantly higher risk of preterm birth and more frequent occurrence of low and very low birth weight of the newborn (30).

The First National Program of ART in Serbia “One free in vitro fertilization (IVF) attempt for 1000 couples” was initiated by the Ministry of Health of the Republic of Serbia (October 2006). Results of this program for the period between March 1, 2007, and March 1, 2009, showed that prematurity, low birth weight, perinatal asphyxia, and systemic infection contribute significantly to the morbidity of an IVF-conceived newborn (31). Beginning in March 2017, Serbia adopted the new Law on Treating Infertility through a procedure of Biomedical Assisted Fertilization (the older law was from 2009). The introduction of this Law had the goal to increase the number of IVF attempts implemented, and the success rate of the procedures performed (32).

Current research suggests that the survival of newborns can be improved by the use of antenatal and perinatal therapies and the reanimation of newborns in specialized healthcare institutions (14). The coverage of pregnant women by antenatal medical care in the first trimester of pregnancy is similar between Serbia (79.8% in 2017) (33) and other high-income countries (around 81.9%) (34). In Serbia, healthcare of newborns is applied in pediatric departments of general hospitals, outpatient clinics, specialized hospitals, and in five major university children’s clinics (35).

It was observed that breastfeeding during the first month of life and the implementation of the Kangaroo Mother Care method lead to a significant reduction in neonatal mortality (36,37). In 2018, the Government of the Republic of Serbia adopted the Decree on the National Program for Support of Breastfeeding, Family, and Developmental Care of Newborns to improve neonatal outcomes, healthcare potential and skill of medical professionals caring for sick children, and children at risk of premature birth (38). The program includes treatment and care based on the principles of individualized developmental care (NIDCAP), application of the Kangaroo Mother Care method, and the early start of natural nutrition (breastfeeding) (39).

Our study has some limitations, which affect the conclusion about the results of the medical care of newborns. A national register of congenital abnormalities does not exist in Serbia (40), and we cannot claim that a decline in neonatal mortality from congenital abnormalities is caused by advanced neonatal care instead of reducing the incidence of congenital abnormalities in newborns.

Data on the rate of premature births, gestational age, and the bodyweight of newborns will contribute to an easier assessment of the effectiveness of antenatal and prenatal care measures for pregnant women and neonatal care of newborns. These data are not available or may be incomplete for some years during the study period, because until 2005 in the Republic of Serbia, there was no prescribed and unique birth application (41).

In conclusion, during the investigated period of 20 years, healthcare in Serbia contributed to the decline in total neonatal mortality, excluding mortality from disorders related to short gestation and low birth weight.

Further investigation and prevention of risk factors of birth prematurity in Serbia, as well as the implementation of the National Program for Support of Breastfeeding, Family, and Development Care, will be required to continue the decline in neonatal mortality.

## Figures and Tables

**Table 1:**
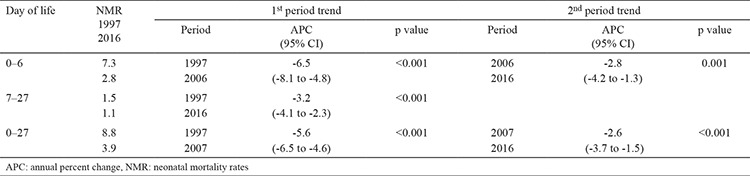
Changes in the early neonatal mortality rates (0–6 days of life) and late neonatal mortality rates (7–27 days of life) per 1000 live births in Serbia, 1997–2016

**Table 2:**
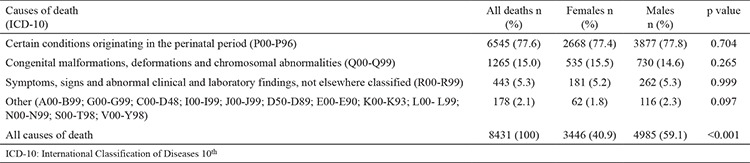
Distribution of neonatal deaths according to causes of death and gender

**Table 3:**
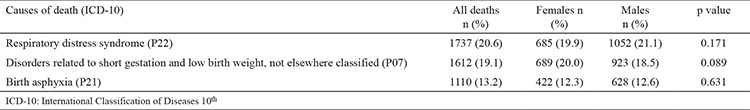
Distribution of the main causes of neonatal deaths according to gender

**Table 4:**
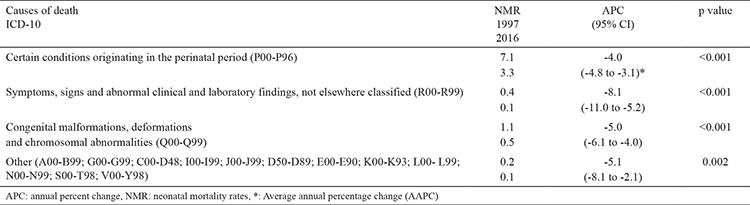
Change in neonatal mortality rates per 1000 live births according to causes of death

**Table 5:**
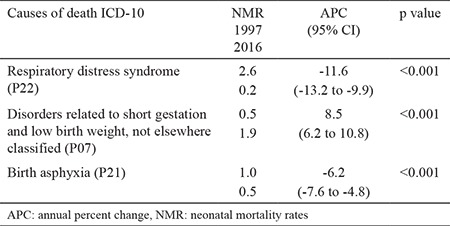
Changes in neonatal mortality rates per 1000 live births according to the three most common causes of death
